# Impact of peripheral conditioning on reperfusion injury following primary percutaneous coronary intervention in diabetic and non-diabetic STEMI patients

**DOI:** 10.1515/med-2025-1175

**Published:** 2025-04-01

**Authors:** Veljko Andric, Radica Zivkovic Zaric, Dusan Andric, Jovan Petrovic, Goran Davidovic

**Affiliations:** Department of Pharmacology and Toxicology, Faculty of Medical Sciences, University of Kragujevac, Kragujevac, Serbia; Department of Internal Medicine, Health Center Raška, Raska, Serbia; Department of Pharmacology and Toxicology, University Clinical Centre Kragujevac, Kragujevac, Serbia; Department of Cardiology, University Children’s Clinic, Belgrade, Serbia; Department of Cardiology and Internal Medicine, Vascular Surgery Clinic, Institute for Cardiovascular Diseases “Dedinje”, Belgrade, Serbia; Faculty of Medical Sciences, University of Kragujevac, Svetozara Markovica 69, Kragujevac, Serbia; Department of Internal Medicine, Faculty of Medical Sciences University of Kragujevac, University Clinical Centre Kragujevac, Kragujevac, Serbia

**Keywords:** reperfusion injury, STEMI, peripheral conditioning

## Abstract

**Background:**

Peripheral conditioning induces transient ischemia, promoting antioxidant production in ischemia-affected tissues, which helps reduce heart reperfusion injury in ST-elevation myocardial infarction (STEMI) patients. This study compares troponin and creatine kinase-MB (CK-MB) levels among STEMI patients with and without remote conditioning.

**Methods:**

This study included 160 patients treated for STEMI at a tertiary care centre. The study protocol involved cyclic inflation and deflation of a blood pressure cuff on the brachial region in four cycles of 5 min each. Markers of myocardial necrosis, CK-MB, and troponin, were monitored before percutaneous coronary intervention (PCI), immediately after, and at 24, 48, and 72 h post-PCI.

**Results:**

CK-MB and troponin levels were significantly lower in non-diabetic patients who underwent remote peripheral conditioning compared to those who did not, with significant reductions observed after PCI (CK-MB: *p* = 0.001; troponin: *p* = 0.033), and at 24 (CK-MB: *p* = 0.015; troponin: *p* = 0.001) and 48 h post-PCI (troponin: *p* = 0.002). In the second phase, no significant differences in CK-MB or troponin levels were found between diabetic patients with and without conditioning. However, a trend toward lower values was noted in the conditioned group. In the third phase, significant reductions in CK-MB (*p* = 0.002) and troponin levels (after PCI: *p* = 0.007; 24 h post-PCI: *p* = 0.045) were observed across all patients who underwent conditioning compared to the control group.

**Conclusion:**

Peripheral pre- and post-conditioning is an economical, simple, and physiological method that effectively prevents and reduces heart damage caused by reperfusion injury, particularly in non-diabetic STEMI patients.

## 1 Introduction

Reperfusion injury is acute tissue damage that occurs suddenly after reperfusion is established over an occluded artery that vascularized the stated tissue. The pathophysiological mechanism accompanying reperfusion injury is based on creating free radicals that overcome the body’s physiological antioxidant protection, causing oxidative stress that further damages the myocardium [1,2].

Remote peripheral conditioning is a procedure that causes transient ischemia, by interrupting the arterial blood flow on a peripheral blood vessel, on one part of the vascular bed, which results in the production of antioxidants by the ischemia-induced tissue [1]. Newly formed antioxidants are transferred through the circulation to other distant, ischemia-affected organs and tissues, where they participate and neutralize reactive oxygen groups and free radicals, thus protecting them from reperfusion injury [1,3]. The concentrations of certain antioxidants that participate in the mentioned protection are super-oxide-dismutase, glutathione peroxidase, vitamin C, and malondialdehyde, and their concentration increases after applying the peripheral conditioning protocol [2,4–6].

This study aimed to find whether artificially induced episodes of ischemia in one upper extremity can affect the reduction of reperfusion injury after percutaneous coronary intervention (PCI) in patients with ST elevation myocardial infarction (STEMI) with or without diabetes.

## 2 Materials and methods

This study included 160 patients treated for STEMI in the coronary care unit of a tertiary care centre. The inclusion criteria were (1) a confirmed diagnosis of acute myocardial infarction (MI) with a STEMI presentation (either first onset or recurrent STEMI), (2) PCI performed within 12 h of symptom onset, (3) administration of bisoprolol upon admission to the coronary care unit, and (4) a thrombolysis in MI flow grade of 0 or 1 in the infarct-related artery.

The exclusion criteria for this study were (1) patients without a diagnosis of STEMI or those diagnosed with STEMI but not undergoing PCI; (2) patients who exceeded the 12 h window from symptom onset to PCI; (3) presence of left bundle branch block on the initial ECG, except in cases with concordant ST elevation ≥1 mm in leads with a positive QRS complex, concordant ST depression ≥1 mm in leads V1–V3, or discordant ST elevation ≥5 mm in leads with a negative QRS complex; (4) right bundle branch block; (5) decompensated heart failure; (6) cardiogenic shock or hemodynamic instability; (7) culprit lesions in the left main coronary artery, high proximal lesions of the left anterior descending artery, or diffuse atherosclerotic disease requiring surgical intervention; (8) prior thrombolytic therapy before PCI; (9) patients with type 2 MI (due to non-atherosclerotic causes of myocardial injury), type 4a MI (periprocedural with troponin elevation >5 times baseline), or type 4b MI (stent thrombosis), as defined by the third universal definition of MI; (10) patients with a life expectancy of less than 1 year from study initiation due to comorbid conditions; (11) prior surgical resolution of ischemia; (12) pregnancy; (13) refusal to sign informed consent; (14) inability to comply with the research protocol; and (15) conditions or deformities preventing proper use of a blood pressure cuff on the upper arm.

The protocol for the study involved cyclic inflation and deflation of a blood pressure cuff placed on the brachial region, in four cycles of 5 min each (5 min of inflation followed by 5 min of deflation). During inflation, the cuff pressure was set to 20 mmHg above the patient’s systolic blood pressure. The deflation procedure involved completely releasing the cuff pressure to a value below the diastolic blood pressure. This protocol was conducted before coronary angiography and PCI (pre-conditioning) and immediately following the PCI procedure (post-conditioning).

The patients were divided into four groups 40 patients each: Group I included patients with STEMI without type 2 diabetes; Group II included STEMI patients with type 2 diabetes; Group III included STEMI patients who underwent remote pre- and post-conditioning but did not have type 2 diabetes; and Group IV included STEMI patients who underwent remote pre- and post-conditioning and had type 2 diabetes.

In these patients, we monitored creatine kinase-MB (CK-MB) and troponin as markers of myocardial necrosis and reperfusion injury size before the PCI procedure, immediately after the PCI procedure, and at 24, 48, and 72 h post-PCI.

We analysed the data by parametric or nonparametric methods. Observed characteristics were expressed as mean values, standard deviation, median, and interquartile range. The Mann–Whitney *U*-test and Wilcoxon signed-rank test were used for continuous nonparametric data, and continuous parametric data were analysed using Student’s *t*-test and paired *t*-test. To assess the strength of the relationship between continuous variables, correlation and regression analyses were performed. Categorical data were analysed using the Chi-square test and Fisher exact test, to determine the statistically significant difference. Significance was set at a two-sided *p* < 0.05. IBM SPSS Statistics 26 (Armonk, New York, USA) was used for the analysis.


**Ethical approval:** The research has been approved by the ethical University Clinical Center Kragujevac; 30.01.2018; No 01/18-455.

## 3 Results

Demographic and clinical data are given in Table 1. In all four groups, males were the predominant sex. As for the age groups the largest number of patients, 90 (56.3%) were aged 41–64, 62 patients (38.8%) belonged to the age group over 65, and 8 patients (5%) were under 40 years old. When it comes to age distribution by group, in all four investigated groups we had the largest number of respondents aged 41–64. We did not find a significant difference in examined parameters between the comparison groups, except in the presence of dyslipidaemia (*p* = 0.005).

**Table 1:** Demographic and clinical characteristics of patients

Factor	Total	Group I	Group II	Group III	Group IV	*p*
	*N* = 160	*N* = 40	*N* = 40	*N* = 40	*N* = 40	
Male sex	111 (69.4)	28 (70)	30 (75)	31 (77.5)	22 (55)	*0.125*
Age (>65 years)	62 (38.8)	12 (30)	15 (37.5)	19 (47.5)	16 (40)	*0.452*
Dyslipidemia	57 (35.6)	10 (25)	21 (52.5)	8 (20)	18 (45)	*0.005*
Smoking	71 (44.4)	19 (47.5)	17 (42.5)	18 (45)	17 (42.5)	*0.964*

Data are given as *n* (%).

Based on the localization of STEMI, the patients were categorized into four groups: anterior, anterior-extended, inferior, and posterior-inferior. Among the entire cohort, the most prevalent type was inferior MI, observed in 67 patients (41.9%). This was followed by anterior-extended infarction in 45 patients (28.1%), posterior-inferior in 28 patients (17.5%), and anterior infarction in 20 patients (12.5%). When comparing the prevalence of inferior infarction across the groups, a slightly higher, but not statistically significant, prevalence of inferior localization was noted compared to the other three groups (*p* = 0.856).

**Figure 1:**
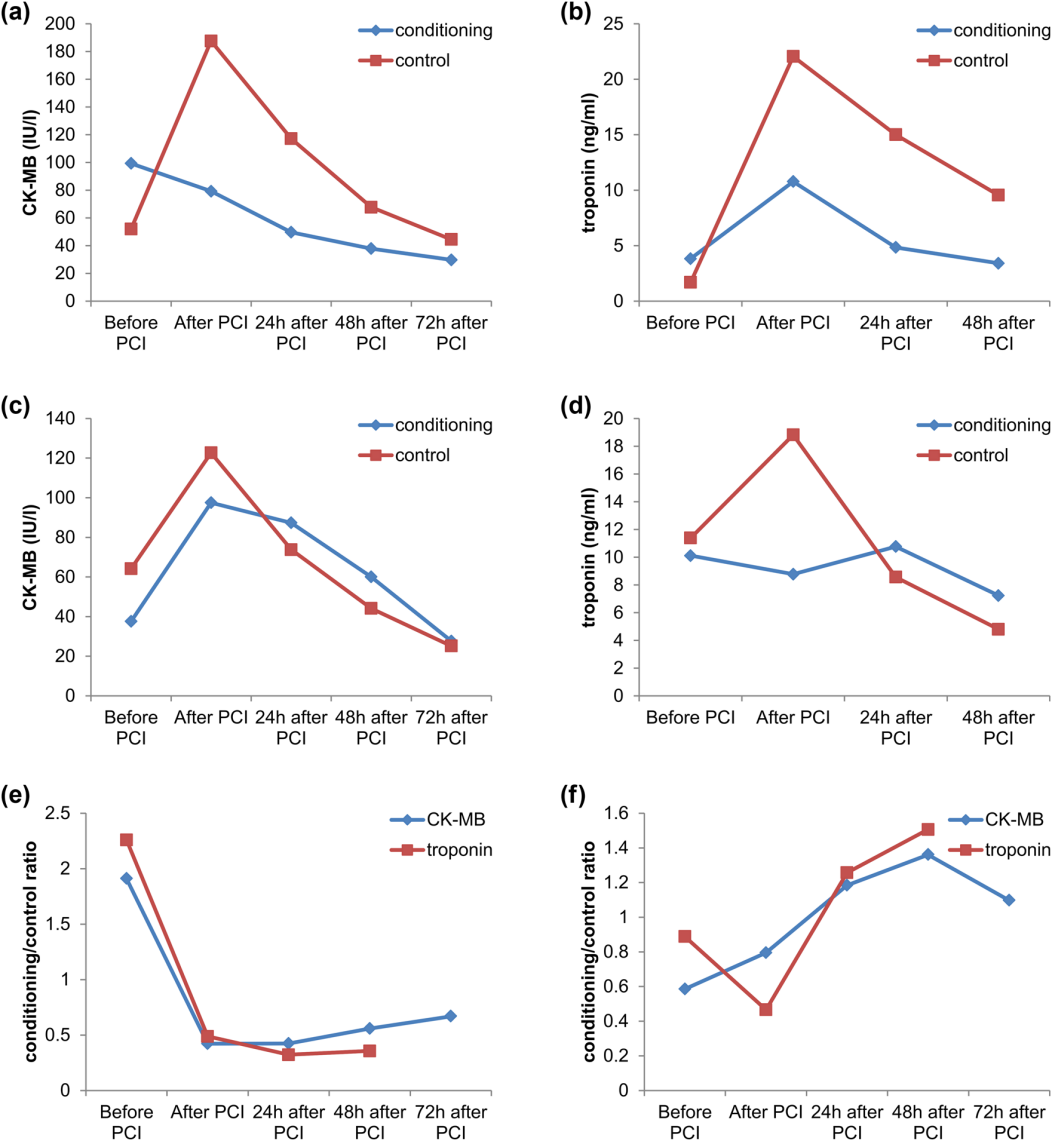
Baseline comparison charts: (a) patients without diabetes mellitus, (b) patients with diabetes mellitus, and (c) CK-MB and troponin values across the control and conditioned groups, regardless of diabetes status.

In the first phase of the study, we compared CK-MB and troponin values between a control group of non-diabetics, non-conditioned patients and a conditioned group of non-diabetic patients who underwent remote peripheral conditioning according to the previously described protocol. Each group consisted of 40 patients. Monitoring CK-MB values at various time intervals revealed a statistically significant decrease in CK-MB levels in the conditioned group after PCI (*p* = 0.001) and 24 h post-PCI (*p* = 0.015), while no significant differences were observed at other time points (Table 2). Similarly, a significant reduction in troponin levels was observed after PCI (*p* = 0.033), 24 h post-PCI (*p* = 0.001), and 48 h post-PCI (*p* = 0.002) in the conditioned group, indicating a beneficial effect of remote peripheral conditioning (Table 2, Figure 1).

**Table 2:** Values of CK-MB and troponin in control and conditioned groups of patients without diabetes

	CK-MB			Troponin		
	Conditioning (IU/L)	Control (IU/L)	*p*	Conditioning (ng/mL)	Control (ng/mL)	*p*
Before PCI	99.30 ± 152.62	51.95 ± 70.41	*0.079*	3.82 ± 9.13	1.69 ± 4.24	*0.184*
After PCI	79.18 ± 126.00	187.60 ± 162.25	*0.001*	10.77 ± 18.56	22.05 ± 27.02	*0.033*
24 h after PCI	49.60 ± 121.39	117.10 ± 121.64	*0.015*	4.84 ± 7.07	15.00 ± 16.31	*0.001*
48 h after PCI	37.83 ± 78.76	67.70 ± 71.69	*0.080*	3.41 ± 5.09	9.55 ± 10.94	*0.002*
72 h after PCI	29.73 ± 62.91	44.43 ± 58.34	*0.282*	—	—	—

CK-MB: creatine kinase-MB; PCI: percutaneous coronary intervention.

In the second phase, we compared these parameters between two additional groups of 40 patients each: the control group, consisting of diabetic patients without conditioning, and the conditioned group, consisting of diabetic patients who underwent conditioning. Although no statistically significant differences were observed in CK-MB or troponin levels between the two groups, a downward trend in these markers was noted in the conditioned group compared to the control group (Table 3).

**Table 3:** Values of CK-MB and troponin in control and conditioned groups of patients with diabetes

	CK-MB			Troponin		
	Conditioning (IU/L)	Control (IU/L)	*p*	Conditioning (ng/mL)	Control (ng/mL)	*p*
Before PCI	37.55 ± 42.78	64.18 ± 74.74	*0.054*	10.11 ± 28.76	11.38 ± 40.52	*0.872*
After PCI	97.48 ± 113.42	122.68 ± 116.34	*0.330*	8.77 ± 9.41	18.82 ± 35.25	*0.085*
24 h after PCI	87.35 ± 111.84	73.78 ± 67.02	*0.512*	10.76 ± 13.45	8.56 ± 9.87	*0.409*
48 h after PCI	60.05 ± 71.26	44.13 ± 32.54	*0.202*	7.23 ± 10.20	4.80 ± 7.35	*0.226*
72 h after PCI	27.63 ± 31.34	25.18 ± 27.79	*0.712*	—	—	—

CK-MB: creatine kinase-MB; PCI: percutaneous coronary intervention.

In the third phase, we analysed the CK-MB and troponin values across the control and conditioned groups, regardless of diabetes status. Each group included 80 patients. Statistically significant differences were observed in CK-MB levels after PCI (*p* = 0.002), as well as troponin levels after PCI (*p* = 0.007) and 24 h post-PCI (*p* = 0.045). A reduction in the levels of these laboratory markers was again noted following remote peripheral conditioning in the conditioned group compared to the control group.

## 4 Discussion

In this study, we observed a significant reduction in CK-MB levels in patients who received pre- and post-conditioning compared to those who did not, immediately after PCI and 24 h post-PCI. No statistically significant differences were found before PCI or at 48 and 72 h post-PCI. A similar pattern was observed with troponin values, which showed significant reductions immediately after PCI, as well as at 24 and 48 h, compared to the control group. Previous research has demonstrated the protective effect of post-conditioning in STEMI patients following angioplasty [7], and this study supports these findings by showing that remote pre- and post-conditioning significantly reduces reperfusion injury after PCI [8]. Other studies have also confirmed the protective effects of peripheral conditioning across various organs, including the brain [9], kidneys [10–12], lungs [13,14], liver [15,16], and skin [4,11].

Systematic review from 2015 also demonstrated the effects of remote ischemic conditioning on cardioprotection among many included studies and it was proven that it significantly decreased troponin and CK MB in blood of patients [17].

Predomination of male gender is in correlation with many other studies in patients with PCI [18]. It was most often in patients younger than 65 years, probably because they see a cardiologist earlier. About half of the patients were smokers which is also in correlation with other studies [19].

Cardiac marker values did not show significant differences between the groups before the conditioning protocol, which is expected given the homogeneity of the study groups concerning factors influencing cardiac marker levels. Statistically significant differences in CK-MB values were noted immediately after post-conditioning and 24 h post-PCI, but not at 48 and 72 h. This timing aligns with the known kinetics of CK-MB concentration, where the conditioning effect is most pronounced during the rising phase and peak concentration period. By 72 h, as CK-MB levels decline, the impact of conditioning is less apparent. Troponin I value showed a similar trend, with significant differences observed immediately after PCI, 24, and 48 h post-PCI. The troponin I value normalizes later than CK-MB, which explains the observed differences between the two markers at 48 h. The second phase of peripheral conditioning effects typically occurs between 12 and 24 h, further supporting the timing of our results.

For diabetic patients, no statistically significant differences in CK-MB and troponin I were found between conditioned and control groups. These findings are consistent with other studies on post-conditioning in both animal models and humans [20,21].

## 5 Conclusion

Peripheral pre- and post-conditioning is an effective, economical, simple, and safe method for preventing and reducing myocardial damage caused by reperfusion injury. This approach can be considered a viable therapeutic option for treating STEMI in non-diabetic patients. However, in diabetic patients with STEMI, the protocol did not show a significant impact on the size of reperfusion injury. Therefore, there is currently no compelling evidence to support its routine use in this population.
